# Symbiotic Plant Peptides Eliminate *Candida albicans* Both *In Vitro* and in an Epithelial Infection Model and Inhibit the Proliferation of Immortalized Human Cells

**DOI:** 10.1155/2014/320796

**Published:** 2014-08-28

**Authors:** Lilla Ördögh, Andrea Vörös, István Nagy, Éva Kondorosi, Attila Kereszt

**Affiliations:** ^1^Institute of Biochemistry, Biological Research Centre of the Hungarian Academy of Sciences, Temesvari Korut 62, Szeged 6726, Hungary; ^2^Institut des Sciences du Végétal, CNRS, Batiment 23, avenue de la Terrasse, 91198 Gif-sur-Yvette Cedex, France

## Abstract

The increasing number of multidrug-resistant microbes now emerging necessitates the identification of novel antimicrobial agents. Plants produce a great variety of antimicrobial peptides including hundreds of small, nodule-specific cysteine-rich NCR peptides that, in the legume *Medicago truncatula*, govern the differentiation of endosymbiotic nitrogen fixing bacteria and, *in vitro*, can display potent antibacterial activities. In this study, the potential candidacidal activity of 19 NCR peptides was investigated. Cationic NCR peptides having an isoelectric point above 9 were efficient in killing *Candida albicans*, one of the most common fungal pathogens of humans. None of the tested NCR peptides were toxic for immortalized human epithelial cells at concentrations that effectively killed the fungus; however, at higher concentrations, some of them inhibited the division of the cells. Furthermore, the cationic peptides successfully inhibited *C. albicans* induced human epithelial cell death in an *in vitro* coculture model. These results highlight the therapeutic potential of cationic NCR peptides in the treatment of candidiasis.

## 1. Introduction

*Candida albicans* is one of the most common opportunistic human fungal pathogens. In healthy human populations, it is a member of the normal flora of the skin, genital, and intestinal mucosa. However,* C. albicans* as well as other* Candida* species (e.g.,* C. parapsilosis* or* C. krusei*) may lead to morbidity and mortality in immunocompromised patients as a consequence of fungal overgrowth and severe cutaneous or systemic infections.* Candida* exhibits fungal dimorphism both* in vitro* and* in vivo*, growing like unicellular budding yeast or filamentous hyphae [1]. The ability for a phenotypic switch from the budding blastospore state to a filament hyphae form is an important factor in its virulence [2].

For the treatment of invasive candidiasis, amphotericin B-based preparations, azoles, and echinocandins are used. In the therapy of mucocutaneous infections (e.g., vaginal infections), azoles are the dominant agents [3]. These antifungal drugs disturb the integrity of the fungal membranes or the fungal cell wall. Amphotericin B binds to sterols in the fungal membranes leading to leakage of monovalent ions and consequently to cytotoxicity. Antifungal triazoles inhibit fungal membrane sterol biosynthesis while echinocandins block the synthesis of (1,3)-*β*-D-glucan, a key component of fungal cell wall [4].

Although the efficiency of anticandidal drugs currently used is satisfactory, there are reports that* C. albicans* has developed resistance against these agents. For example, azole-resistant strains emerged in which mutations affected the drug target Erg11p involved in ergosterol biosynthesis [4, 5]. Similarly, resistance against amphotericin and echinocandins is rare; however, it does occur if levels of the plasma membrane ergosterol and the cell wall (1,3)-*β*-D-glucan are reduced by mutations in the* ERG3* and* GSC1* genes, respectively [4]. Although the appearance of resistant strains is not a serious problem when compared to the spread of multidrug-resistant bacterial pathogens, the development of novel antifungal agents is still desired as a precaution.

Since the occurrence of antibiotic-resistant superbugs resulting from the widespread use of conventional antibiotics is emerging, numerous studies have focused on the use of antimicrobial peptides (AMPs) as therapeutic agents [6, 7]. AMPs represent ancient host defence effector molecules that are present in all organisms across the evolutionary spectrum. AMPs produced by the host innate immune system have broad-spectrum and rapid killing activities against a wide range of microorganisms, including fungi [8]. AMPs are usually cationic and amphipathic peptides that are able to interact with the negatively charged microbial membranes. Their killing action can be based on membrane permeabilization and cell lysis, as well as their interactions with cytoplasmic targets. As multiple targets likely exist, the development of resistance against AMPs seems less probable than in the case of classical antibiotics [9, 10].

In contrast to the relatively low numbers of AMPs in animals, plants have evolved an extreme richness of AMPs that are often present in specific plant organs, such as the roots, seeds, flowers, stems, and leaves. The* Arabidopsis* genome contains 317 small genes coding for cysteine-rich defensin peptides [11]. However, AMP-like peptides not only act as defense effectors but also can function in symbiosis controlling the cell number and differentiation of endosymbiotic bacteria. This has been discovered in the* Medicago truncatula*-*Sinorhizobium meliloti* symbiosis, which leads to the development of root nodules where bacteria inside the plant cells mature progressively to nitrogen-fixing bacteroides. Differentiation of the bacterium partner is irreversible and governed by host AMP-like symbiotic peptides, which are targeted via the secretory pathway to the endosymbiotic bacteria [12]. The largest group with up to 500 different members is the nodule-specific cysteine-rich (NCR) peptide family. NCR peptides contain a relatively conserved signal peptide and a highly diverse mature peptide composed of 30–50 amino acids with conserved positions of four or six cysteines. Due to the high sequence diversity, the isoelectric point (pI) of the peptides ranges from 3.2 to 11.2. The combined action of different NCR peptides results in the definitive loss of cell division ability and in the development of noncultivable polyploid endosymbionts with increased membrane permeability.* Ex planta*, several cationic NCR peptides of* M. truncatula* proved to be potent killer of various Gram-negative and Gram-positive bacteria [12, 13].

In this study, we investigated whether NCR peptides with antibacterial activity against human and plant pathogenic bacteria exhibit anticandidal activity and, if so, whether they are cytotoxic for human cells. The anticandidal activity of the peptides was tested by treatment of fungal cells with synthetic NCR peptides and in an* in vitro* infection system where vaginal epithelial cells were cocultured with* C. albicans*.

## 2. Materials and Methods

### 2.1. Strains, Media, and Peptides

The* C. albicans* strains WO-1 and Sc5314 were maintained on YPD medium (1% (w/v) yeast extract, 2% (w/v) peptone, 2% (w/v) dextrose, 2% (w/v) agar; Beckton, Dickinson and Company). Hyphal growth was induced in complete keratinocyte medium (CKM; Life Technologies) without serum. The effect of the NCR peptides on free-living fungi was investigated either in PBgluc buffer (1 mM KH_2_PO_4_, 3 mM Na_2_HPO_4_ × 7H_2_O, 5% glucose, pH = 7.0) or in low-salt fungal medium (LSM) composed of 5 mM K_2_HPO_4_, 100 *μ*M MgSO_4_, 10 *μ*M FeCl, 0.2 *μ*M CoCl_2_, 0.2 *μ*M CuSO_4_, 4 *μ*M Na_2_MoO_4_, 1 *μ*M H_3_BO_3_, 0.2 *μ*M KI, 1 *μ*M ZnSO_4_, 0.2 *μ*M MnSO_4_, 2% glucose, 0.2% asparagine, 40 *μ*g/mL methionine, 4 *μ*g/mL myo-inositol, 0.4 *μ*g/mL biotin, 2 *μ*g/mL thiamine-HCl, and 0.4 *μ*g/mL pyridoxine-HCl [14].

The immortalized human vaginal epithelial cell line PK E6/E7 [15] was cultured in serum-free CKM supplemented with 0.005 *μ*g/mL recombinant epidermal growth factor, 50 *μ*g/mL bovine pituitary extract, L-glutamine, and antibiotic/antimycotic solution (Life Technologies) in a CO_2_ thermostat at 37°C [16]. Cells at 60–70% confluence were used in subsequent experiments.

Mature (without the signal peptide) and N-terminally fluorescein isothiocyanate (FITC) labeled NCR peptides (Table 1) were chemically synthesized (>95% purity, Proteogenix, France) and dissolved in MilliQ water and were diluted in LSM or PBgluc. Biological activity of the NCR peptides was tested in LSM or PBgluc.

### 2.2. Antifungal Assays

The antifungal activity of the NCR peptides against* C. albicans* strains and other* Candida* species was determined* in vitro* using 96-well flat bottom microtiter plates (Sarstedt). Fifty microliters of dilutions from each peptide (final concentrations in the assays ranged from 2.5 to 100 *μ*g/mL) was added to 50 *μ*L of 10^4^ cells mL^−1^ and 10^5^ cells mL^−1^ in LSM. The plates were incubated at 30°C and the peptide concentrations that prevented the germination of yeast cells were determined by microscopic analysis and measurement of absorbance at 600 nm with a microtiter plate reader (FLUOstar OPTIMA; BMG Labtech) after 24 and 48 hours. Minimal inhibitory concentration (MIC) was determined as the lowest concentration of the peptide at which there was no visible and measurable growth after 24 hours of incubation. To determine whether the antifungal effect was fungistatic or fungicidal, 10 *μ*L from each treatment was dropped onto solid YPD plate after 3 and 24 hours. Plates were incubated at 30°C for 48 hours and developing colonies were observed. Minimal fungicidal concentration (MFC) was defined where no fungal growth could be observed.

To investigate the effect of the peptides on the pseudohyphal form, its growth was induced by incubating* C. albicans* cells in serum-free CKM without antibiotic/antimycotic solution for 3 hours at 30°C in a 96-well plate; then the medium was removed and NCR peptides diluted in LSM were added. After 2 hours of treatment, the morphological changes of fungal hyphae were investigated by microscopy.

### 2.3. Fluorescent and Confocal Microscopy

*C. albicans* WO-1 cells were labeled with 5 *μ*g/mL of the FM4-64 membrane stain (Life Technologies) and 0.5 *μ*g/mL of the DNA stain 4′,6-diamidino-2-phenylindole (DAPI, Sigma) for 15 minutes after 2 hours of peptide treatment. Images were captured with an Axio Observer Z.1 (Zeiss) fluorescent microscope. For studying the interaction of peptides with the fungus, an overnight* C. albicans* WO-1 culture was diluted in LSM and treated with FITC-conjugated peptides (at the sublethal concentration of 5 *μ*g/mL) for 3 hours at 30°C. Cells were then centrifuged for 4 minutes at 600 rpm and the unbound peptides were removed by gentle pipetting of the supernatant and cells were resuspended in LSM. The cell suspension was dropped onto a coverslip and images were taken with an Olympus FluoView FV1000 confocal laser-scanning microscope.

### 2.4. Cell Permeability Assay

Cell permeability was monitored by the release of the intracellularly accumulated dye calcein. Overnight* C. albicans* WO-1 cells were washed twice with and resuspended in 10 mM potassium phosphate buffer (pH = 7.0) at a concentration of 10^7^ cells mL^−1^. Cells were loaded with acetomethoxy-calcein at a final concentration of 5 *μ*M for 2 hours at room temperature. Cells were washed three times to remove the extracellular dye and then 100 *μ*L of the suspension was transferred to FIA black 96-well plates. NCR peptides were then added at a concentration of 50 mg/L, and the release of calcein (formed by the cells' esterases) was measured by its fluorescence in a microtiter plate reader (FLUOstar OPTIMA) at excitation and emission wavelengths of 485 nm and 530 nm, respectively. Calcein efflux was calculated as previously reported [17].

### 2.5. Analysis of the Cytotoxic Effect of NCR Peptides on Human Epithelial Cells

*MTT Assay*. PK E6/E7 vaginal epithelial cells were seeded in the wells of a 96-well plate at a density of 1 × 10^4^ cells per well in a final volume of 100 *μ*L. CKM was then aspirated and 100 *μ*L of the appropriate peptide diluted in PBgluc was added. After 3 hours of incubation at 37°C, the peptide solution was removed, and the cells were let to regenerate in CKM for 24 hours. Then 5 mg/mL 3-[4,5-dimethylthiazol-2-yl]-2,5-diphenyltetrazolium bromide (MTT) was added to each well and the plate was placed to 37°C for 4 hours. The amount of formazan formed by the activity of the mitochondrial succinate dehydrogenase of living cells was measured by using a microtiter plate reader (FLUOstar OPTIMA) at the wavelength of 560 nm [18].

*Real-Time Cell Analysis* (*RTCA*). 7 × 10^4^ PK E6/E7 cells per well were seeded in 96-well E-plates (ACEA Biosciences) in which the bottoms of the wells were covered with microelectrodes and the epithelial cells were allowed to attach to the bottom of the wells for 20 hours. CKM was then aspirated and the cells were treated with 100 *μ*L of the peptides diluted in PBgluc. After 3 hours of incubation at 37°C, the peptide suspension was removed and 200 *μ*L of CKM was added to the cells. Subsequent real-time measurements of impedance were done with the xCELLigence System RTCA HT Instrument (ACEA Biosciences) for 72 hours. The cell index at the beginning of the measurement after treatment was considered as 100% and values measured at 40 hours were expressed (see Supplementary Figure 1in Supplementary Materials available online at http://dx.doi.org/10.1155/2014/320796).

10 *μ*g/mL of primycin, a macrolide lactone antibiotic complex [19] that rapidly kills PK cells, was used as a control for cytotoxicity in both assays.

### 2.6. The Effect of NCR Peptides on Vaginal Epithelial Cell:* C. albicans *Coculture

PK E6/E7 cells were seeded in 96-well E-plates as described above for RTCA. On the next day, an overnight culture of* C. albicans* WO-1 was diluted to OD = 1 and let to grow until OD = 1.5–2. The fungal cell suspension was diluted in antibiotic/antimycotic-free CKM and was added to the human cells with a multiplicity of infection (MOI) of 5. After 3 hours of coculture at 37°C, CKM medium was replaced with NCR peptides in PBgluc. After 3 hours of incubation, peptides were removed and 200 *μ*L of fresh antibiotic/antimycotic-free CKM was added. Subsequent measurements were performed as described above.

### 2.7. Data Presentation and Statistics

MTT assays and impedance measurements were performed in at least four technical parallels and two biological replicates; all the data are presented as mean ± standard deviation. Significance was calculated with Newman-Keuls post hoc test using the GraphPad Prism version 5 for Windows and a probability (*P*) value of less than 0.05 was considered significant.

## 3. Results

### 3.1. Anticandidal Activity of the NCR Peptides

Since cationic defensin-like NCR peptides have been shown to be active against a wide range of Gram-negative and Gram-positive bacteria, including animal and plant pathogens, 19 NCR peptides covering the isoelectric point (pI) spectrum of the peptide family (Table 1) were investigated for anticandidal activity in microdilution plate assays. In serum-free RPMI1640 (GIBCO), NCR peptides did not have any anticandidal activity (data not shown) which was not surprising as the concentration of NaCl (>100 mM) and the presence of Ca^2+^ and Mg^2+^ ions had been previously shown to inhibit their bactericidal effect [12] and are known to block AMP activities [20, 21]. Hence, the NCR activity tests were performed in LSM medium that had been previously used successfully to measure antifungal activity of defensins. Likewise, the defensins [22], only cationic NCR peptides, particularly with pI > 9.5 (NCR192, NCR137, NCR147, NCR280, NCR183, NCR247, NCR044, NCR030, and NCR335) exhibited anticandidal activity against the yeast form of growing* C. albicans* strains WO-1 and Sc 5314 (Table 2). The MIC values of these peptides (Table 2) ranged from 10 to 50 *μ*g/mL (1.42–10.5 *μ*M), which is comparable to the values observed for amphotericin B (1.69 *μ*M). These cationic NCR peptides were also active against other* Candida *species, such as* C. glabrata*,* C. parapsilosis,* or* C. krusei,* at the same concentration range as for* C. albicans* (10 to 50 *μ*g/mL).

In order to determine whether the cationic peptides are fungistatic or fungicidal,* C. albicans* cell suspensions were treated for 3 and 24 hours with different concentrations of the peptides, and 10 *μ*L of the cultures was then incubated on solid YPD medium for 48 hrs. Anionic NCR peptides had no effect on fungal growth. In contrast, cationic NCR peptides exhibited rapid fungicidal activity* in vitro*. The MFC values of the nine cationic NCR peptides with pI > 9.5 ranged from 6.25 to 25 *μ*g/mL (Table 3) and were comparable with the MIC values obtained with the microdilution assay.

**Figure 1:**
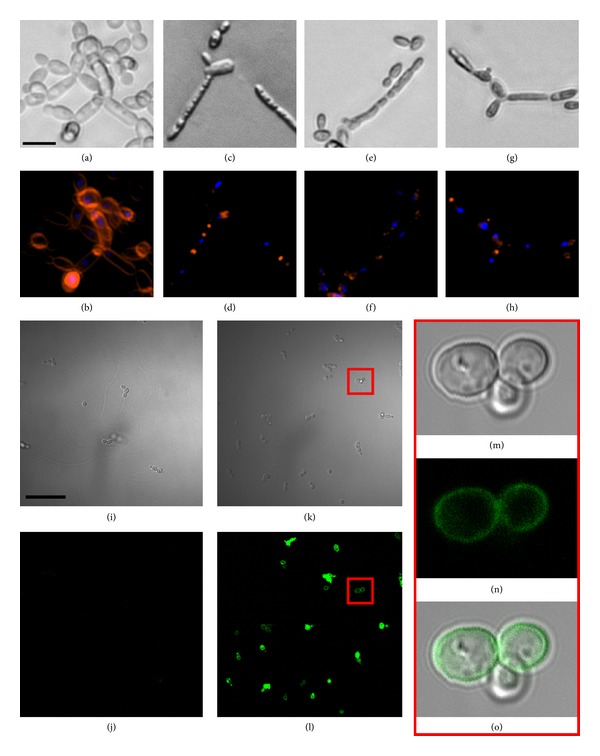
NCR provoked morphological changes and localization of fluorescently labeled NCR peptides in* C. albicans* cells and pseudohyphae. ((a)–(h)) Pseudohyphal* C. albicans* WO-1 cells were either mock treated (a), (b) or treated with NCR335 (c), (d); NCR247 (e), (f); NCR192 (g), (h) peptides at 25 *μ*g/mL concentration for 2 hours. Differential interference contrast (DIC) ((a), (c), (e), and (g)) and fluorescent ((b), (d), (f), and (h)) images obtained after FM4-64 (cell membrane; red) and DAPI labeling (DNA; blue). ((i)–(o)) Planktonic cells were treated with 5 *μ*g/mL of FITC-conjugated NCR035 (i), (j) or NCR247 (k)–(o) and the localization of the peptides was monitored with confocal microscopy 3 hours after the treatment. DIC ((i), (k), and (m)) and fluorescent images ((j), (l), and (n)); (o) merged image of (m), (n). Scale bars: (a) 20 *μ*m, (i) 100 *μ*m.

The morphological changes associated with the loss of viability were investigated with fluorescent microscopy. In the presence of cationic NCR peptides at a concentration of 25 *μ*g/mL, a decreased size of the treated cells was detectable in the growing pseudohyphae after only 2 hours of treatment (Figures 1(c), 1(e), and 1(g)). Moreover, the noncontinuous and highly reduced membrane labeling by the FM4-64 membrane stain in these cells was indicative of severe membrane damages (Figures 1(d), 1(f), and 1(h)).

### 3.2. Localization of NCR Peptides in* C. albicans* Cells

Although disruption of cell membranes is considered as a general action of cationic AMPs, they might exert other effects by entering the target cells and inhibiting DNA, RNA, or protein synthesis [22]. Therefore, the cell penetration properties and the localization of the peptides in* C. albicans* cells were also investigated. For this purpose, FITC-conjugated NCR247 peptide was synthesized; the conjugated peptide retained comparable anticandidal activity to the unconjugated form (data not shown). Similarly and as a control, the inactive NCR035 peptide was also labeled with FITC.* C. albicans* WO-1 cells were treated with the FITC-labeled peptides and the localization of fluorescence signals was observed with confocal microscopy. As expected, NCR035 did not show interaction with the negatively charged fungal membranes and did not label the* C. albicans* cells (Figures 1(i) and 1(j)), in keeping with its lack of anticandidal activity (Table 2). In contrast, the FITC-labeled NCR247 at sublethal concentration (5 *μ*g/mL) was primarily localized to the fungal cell membrane, but it was also detected intracellularly (Figures 1(l), 1(n), and 1(o)). These data suggest that the fungicidal effect of cationic NCR peptides might rely on multiple targets, not only predominantly on the fungal plasma membrane, but also intracellularly.

### 3.3. Cationic NCR Peptides Affect* C. albicans* Membrane Permeability

**Figure 2:**
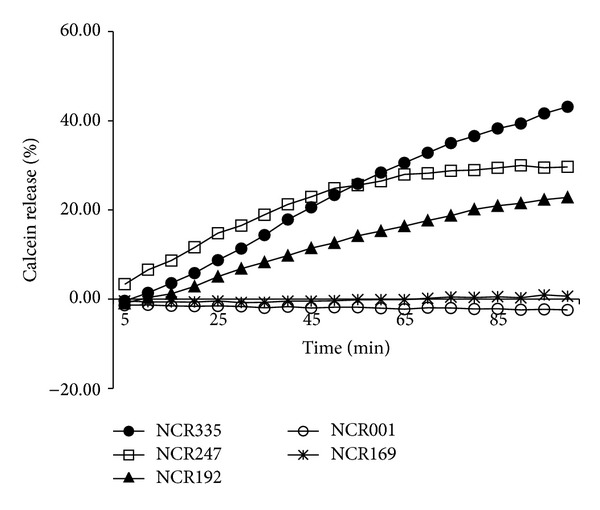
Time course of calcein release from NCR-treated* C. albicans* WO-1 cells. Calcein-loaded cells were treated with the peptides at 50 *μ*g/mL concentration and the fluorescence of the released calcein was measured with a microtiter plate reader (excitation 485 nm, emission 530 nm).

Membrane localization of cationic NCR peptides as well as aberration of the membrane staining in* C. albicans* cells treated with a cationic NCR peptide indicated the potential of membrane permeabilization. This was tested in calcein-loaded* C. albicans* WO-1 cells by measuring the release of the fluorescent dye from the* Candida* cells after the addition of NCR peptides at a concentration of 50 *μ*g/mL (Figure 2). As expected, the cationic NCR peptides (NCR335, NCR247, and NCR192) caused significant dye efflux (43.12 ± 13.05%, 29.65 ± 12.6%, and 22.82 ± 11.16%, resp.) whereas weak cationic (NCR169) and anionic (NCR001) peptides provoked no calcein release. These data confirm that NCR peptides at the MIC or higher concentrations can disrupt the integrity of the fungal membranes.

### 3.4. Determination of the Cytotoxicity of NCR Peptides on Human Cells

An antimicrobial agent cannot be a successful candidate for subsequent use in healthcare if it exhibits toxic activity against human cells. As a consequence, in order to determine whether NCR peptides are cytotoxic against human cells, two cell types, vaginal epithelial cells and keratinocytes (data not shown because of the similar results) that are the targets of* Candida* infections, were tested. The cytotoxicity was measured with cell viability/proliferation assays. The MTT assay provides information on the metabolic state of the cells by measuring the activity of mitochondrial dehydrogenases via the conversion of the tetrazolium dye MTT to formazan. The RTCA assay provides real-time, quantitative information about the number of the living, attached cells by measuring electrode impedance.

The NCR peptides had no toxicity in any of the tested media, not even in serum-free human cell media, most probably because of their inactivation by the elevated concentrations of salts and divalent cations. The LSM fungal medium was inappropriate since human cells died rapidly in this medium already in the absence of NCR peptides (data not shown). In contrast, in isotonic phosphate buffered saline containing 5% glucose (PBgluc), the human cells could survive and the NCR peptides retained their antifungal activity.

**Figure 3:**
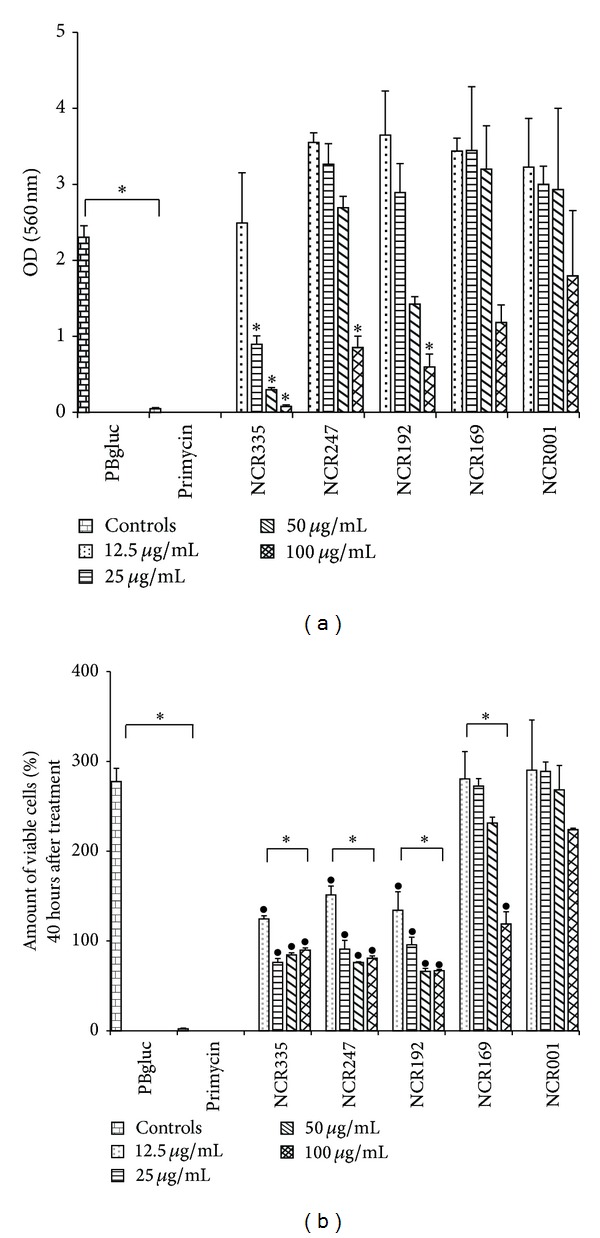
Viability/metabolic activity (a) and proliferation ability (b) of PK E6/E7 vaginal epithelial cells after NCR treatment. (a) The amount of formazan formed after treatment was measured by the absorbance at 560 nm. (b) The relative amount of viable, attached cells 40 hours after treatment was measured by RTCA. The number of cells that were attached when peptides were replaced with CKM after the treatment was considered as 100%. PBgluc: mock treatment; primycin: cytotoxic control. *Significant (*P* < 0.0001) difference between the connected treatments. ^•^The observed cell number significantly (*P* < 0.0001) differs from that of the PBgluc and primycin controls.

In the MTT assay, cationic NCR peptides at MICs were nontoxic for the human cells and only high concentrations (e.g., >25 *μ*g/mL NCR335; 100 *μ*g/mL NCR247; and 100 *μ*g/mL NCR192) provoked cell death, unlike primycin used for treatments of skin infections (resulting from burns) [23] which already at 10 *μ*g/mL concentration killed epithelial cells with about 90% efficiency (Figure 3). Importantly, the antifungal concentration range of cationic NCR peptides that efficiently killed* C. albicans* (<25 *μ*g/mL) affected only slightly the viability of the human cells (Figure 3(a)). On the other hand, when the cell proliferation ability was measured with RTCA, the surviving cells showed a highly reduced cell proliferation at 12.5 *μ*g/mL concentration and at higher concentrations they could not maintain their proliferation ability (Figure 3(b)). The anionic NCR001 peptide had no negative effect on the viability and proliferation of the human cells, while the less cationic NCR169 peptide showed toxicity only at 100 *μ*g/mL concentration (Figure 3).

### 3.5. NCR Peptides Successfully Inhibit* C. albicans* Induced Killing of Vaginal Epithelial Cells* In Vitro*

**Figure 4:**
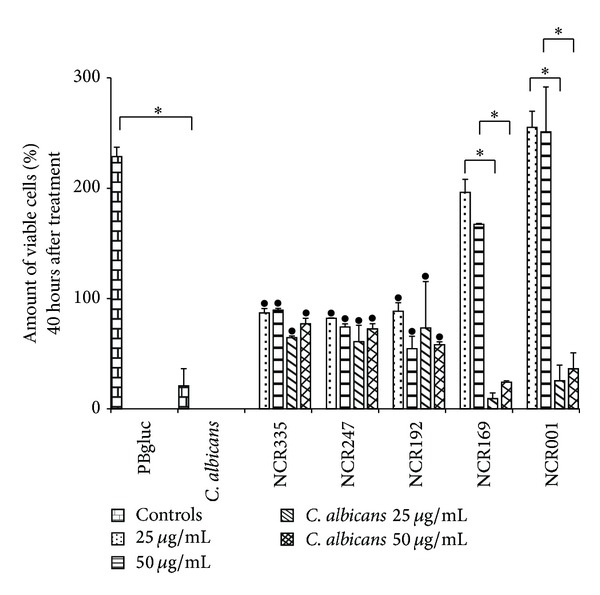
Effects of the NCR peptides on PK E6/E7 vaginal epithelial cell—*C. albicans* coculture. The relative amount of viable, attached cells 40 hours after treatment was measured by RTCA as in Figure 3. 25 and 50 mg/L indicate treatments of the human cells only with peptides;* C. albicans* 25 *μ*g/mL and* C. albicans* 50 *μ*g/mL show the result from treatments of the cocultures with peptides at 25 *μ*g/mL or 50 *μ*g/mL concentrations, respectively. *Significant (*P* < 0.0001) difference between the connected treatments. ^•^The observed cell number significantly (*P* < 0.0001) differs from that of the mock (PBgluc) and infected (*C. albicans*) controls.

Low concentrations of cationic NCR peptides efficiently eliminated* C. albicans* without affecting the viability of vaginal epithelial cells. It is the hallmark of* C. albicans* that it adheres to and kills human cells, mainly by invasive hyphal growth [24]. To test if NCR peptides are able to prevent* C. albicans* induced killing of vaginal epithelial cells, an* in vitro* coculture model was used in which human cells were infected with* C. albicans* WO-1 at an MOI of 5 and incubated for 3 hours to allow the formation of filaments around the human cells before the treatment of the cultures with the NCR peptides. After incubation of the cells for 40 hours, the fungus induced epithelial cell death, which could be prevented by the treatment with the cationic peptides NCR335, NCR247, and NCR192 at both 25 and 50 *μ*g/mL concentrations (Figure 4). The less cationic NCR169 and the anionic NCR001 peptides could not eliminate* C. albicans* cells; these latter two NCR peptides were unable to prevent vaginal epithelial cell death.

Taken together our data suggest that cationic NCR peptides can efficiently kill* C. albicans* at the concentrations that are nontoxic for the human epithelial cells, making them potential anti-*Candida* drug candidates for use in healthcare.

## 4. Discussion

The present study reveals that cationic NCR peptides with a pI above 9, that had previously been shown to have bactericidal effect on the symbiotic rhizobia as well as Gram-negative and Gram-positive pathogenic bacteria* in vitro* [12, 13], are broad-spectrum antimicrobial agents. This subgroup of defensin-like molecules proved to be effective against all tested* Candida* species including* C. albicans*, one of the most common opportunistic human fungal pathogens, via inhibition of growth; more precisely, they affected survival of both the yeast and the filamentous forms of the fungus at a concentration range from 1.5 to 10.5 *μ*M. These values are very similar to the MIC of amphotericin B (~1.7 *μ*M) indicating that,* in vitro*, these cationic NCR peptides are as effective as the conventional anticandidal chemical.

It is well known that several defensins exert antimicrobial activity against bacteria and/or fungi via the permeabilization of target cell membranes [25]. The accumulation of fluorescently labeled cationic NCR247 also indicates that the negatively charged* Candida* cell membranes are primary targets for NCR peptides. Nevertheless, based on the presence of intracellular fluorescence labelling, cytosolic targets cannot be excluded either. The active cationic NCR peptides caused severe morphological changes in the fungal cells manifested by dramatic destruction of the cell membrane structure after only 2 hours of treatment. This membrane damage was also supported by the release of calcein from the NCR-treated form. Although the majority of cationic antimicrobial peptides in animals induce membrane permeabilization via electrostatic interaction with plasma membrane phospholipids [26], plant defensins can achieve the same effect through specific interaction with high affinity binding sites on fungal cells [27]. For example, RsAFP2 can interact with the fungal membrane lipid, glucosylceramide (GlcCer), thereby arresting the fungal growth and leading to cell death [28, 29]. The fungicidal activity of the cationic NCR peptides on* Candida glabrata*, a species that is unable to synthesize GlcCer, however, suggests that GlcCer may not be the target of these peptides; thus, it is possible that they achieve membrane permeabilization in the same manner as animal defensins.

Although the chemical composition of the bacterial, fungal, and mammalian membranes can vary to a high extent, their major components are the phospholipids that have a negatively charged phosphate group in the hydrophilic head, which is targeted by membrane-disrupting cationic peptides. As a therapeutic agent should not be toxic to the patients, the cytotoxicity of selected NCR peptides on different immortalized human cell lines was investigated. In contrast to the macrolide antibiotics primycin, even the most potent cationic peptides were not cytotoxic at concentrations that were effective against bacteria and fungi, thus indicating that their membrane-damaging activity might not target mammalian phospholipids and membranes. On the other hand, they affected the proliferation of immortalized human cells; however, it remains to be elucidated whether both DNA synthesis and cell division, or only the cytokinesis, were inhibited similarly to the bacterial cells when treatment with sublethal concentrations of cationic NCR peptides resulted in increased (up to 16 copies) DNA content [12]. The localization and the cellular targets of the peptides are also to be determined. These results might now open new research directions; if the molecular mechanism of the cell division inhibition can be revealed and the targeted delivery of the active cationic peptides to the cancer cells, as well as the maintenance of their activity during administration, can be achieved, these peptides might arrest the growth of tumors.

The active cationic NCR peptides not only could eliminate the yeast form of the fungi, but also were able to prevent the growth and the subsequent human cell killing activity of the hyphal form in an* in vitro* coculture model. This indicates that, with proper formulation that helps to maintain the activity of the peptides on the human epithelial surface, they might be effective drugs for the treatment of candidiasis.

## 5. Conclusions

Most of the tested cationic NCR peptides showed both bactericidal and fungicidal activities; thus, they may be considered as broad-spectrum AMPs. In contrast to the antibiotic complex primycin, NCR peptides showed toxicity on the epithelial cells at much higher concentrations than their MFC/MIC against* Candida* species. The NCR peptide concentrations that efficiently eliminated the fungi (this work) and bacteria [12, 13] did not affect the survival of the human cells but did restrict their proliferation. These features qualify the peptides to become true candidates for the treatment of microbial infections. It must be noted, however, that salts and components of the serum inhibit the activity of NCR peptides similarly to other AMPs. Therefore, NCR peptides or their derivatives could be ideal antimicrobial agents for topical treatments while their salt and serum sensitivity may limit their utilization as systemic compounds or require their special delivery to the infected tissues or even to cancer cells.

## Supplementary Material

Supplementary Figure 1: Representative output of an RTCA experiment.

## Figures and Tables

**Table 1:** List of NCR peptides used in this study. Length: number of amino acids; MW: molecular weight in Da; pI: isoelectronic point.

Peptide	Sequence of the mature peptide	Length	MW	pI
NCR168	YPFQECKVDADCPTVCTLPGCPDICSFPDVPTCIDNNCFCT	41	4476	3.61
NCR095	ELVCDTDDDCLKFFPDNPYPMECINSICLSLTD	33	3770	3.62
NCR051	EEDIGGHLECVEDEDCMEESCPIFSVHKCKNSGCECDEMFR	41	4684	4.14
NCR235	DTDPFAFCIKDSNCGQDLCTSPNEVPECRLLKCQCIKS	38	4223	4.53
NCR224	KDLPFNICEKDEDCLEFCAHDKVAKCMLNICFCF	34	3987	4.65
NCR001	AFERTETRMLTIPCTSDANCPKVISPCHTKCFDGFCGWYIEGSYEGP	47	5263	5.01
NCR084	FATGMPCKTDKECPNTSTHKYKCINDDCFCFYIYWPLGNSLV	42	4856	6.71
NCR169	EDIGHIKYCGIVDDCYKSKKPLFKIWKCVENVCVLWYK	38	4565	8.45
NCR055	VNDCIRIHCKDDFDCIENRLQVGCRLQREKPRCVNLVCRCLRR	43	4759	9.21
NCR035	SFLGTFISSCKRDKDCPKLYGANFRCRKGTCVPPI	35	3910	9.42
NCR192	MKNGCKHTGHCPRKMCGAKTTKCRNNKCQCVQL	33	3708	9.54
NCR137	MTLRPCLTDKDCPRMPPHNIKCRKGHCVPIGKPFK	35	4018	9.7
NCR147	IYFPVSRPCITDKDCPNMKHYKAKCRKGFCISSRVR	36	4249	9.76
NCR280	MRVLCGRDGRCPKFMCRTFL	20	2390	9.8
NCR183	ITISNSSFGRIVYWNCKTDKDCKQHRGFNFRCRSGNCIPIRR	42	4979	10.1
NCR247	RNGCIVDPRCPYQQCRRPLYCRRR	24	3009	10.15
NCR044	AFIQLSKPCISDKECSIVKNYRARCRKGYCVRRRIR	36	4318	10.32
NCR030	AFLPTSRNCITNKDCRQVRNYIARCRKGQCLQSPVR	36	4197	10.37
NCR335	RLNTTFRPLNFKMLRFWGQNRNIMKHRGQKVHFSLILSDCKTNKDCPKLRRANVRCRKSYCVPI	64	7736	11.22

**Table 2:** Minimal inhibitory concentration (*μ*g/mL) of the NCR peptides against *C. albicans* strains WO-1 and Sc5314 after 24 hours of treatment *in vitro*.

Peptide	WO-1	Sc5314
NCR168	—	—
NCR095	—	—
NCR051	—	—
NCR235	—	—
NCR224	—	—
NCR001	—	—
NCR084	—	—
NCR169	—	—
NCR055	50 (±4)	50 (±3)
NCR035	—	—
NCR192	10 (±2)	12.5 (±1)
NCR137	20 (±2)	25 (±2)
NCR147	19 (±9)	12.5 (±2)
NCR280	19 (±6)	25 (±3)
NCR183	19 (±6)	12.5 (±1.5)
NCR247	14 (±9)	25 (±2)
NCR044	11 (±3)	12.5 (±1)
NCR030	15 (±7)	25 (±5)
NCR335	11 (±1)	12.5 (±2.5)

—: no growth inhibition was observed.

**Table 3:** Minimal fungicidal concentration (*μ*g/mL) of the active NCR peptides against *C. albicans* WO-1 after 3 or 24 hours of treatments.

Peptide	3 h	24 h
NCR192	8 (±3,5)	9 (±4)
NCR137	20 (±2.5)	25 (±3)
NCR147	8 (±6.5)	9 (±4)
NCR280	10 (±2)	8 (±3)
NCR183	16.5 (±12)	15.5 (±13)
NCR247	14 (±10)	16.5 (±13)
NCR044	11 (±2)	6.25 (±1)
NCR030	12.5 (±2)	16.5 (±5)
NCR335	7 (±1)	8 (±2)
