# Supplementary material for: Symbiotic Plant Peptides Eliminate Candida albicans Both In Vitro and in an Epithelial Infection Model and Inhibit the Proliferation of Immortalized Human Cells
Source: Biomed Res Int. 2014 Aug 28;2014:320796. doi: 10.1155/2014/320796 (PMC4163382; doi:10.1155/2014/320796)
Supplement: Supplementary file 1 — Supplementary Figure 1: Representative output of an RTCA experiment. [file 320796.f1.pdf]

A

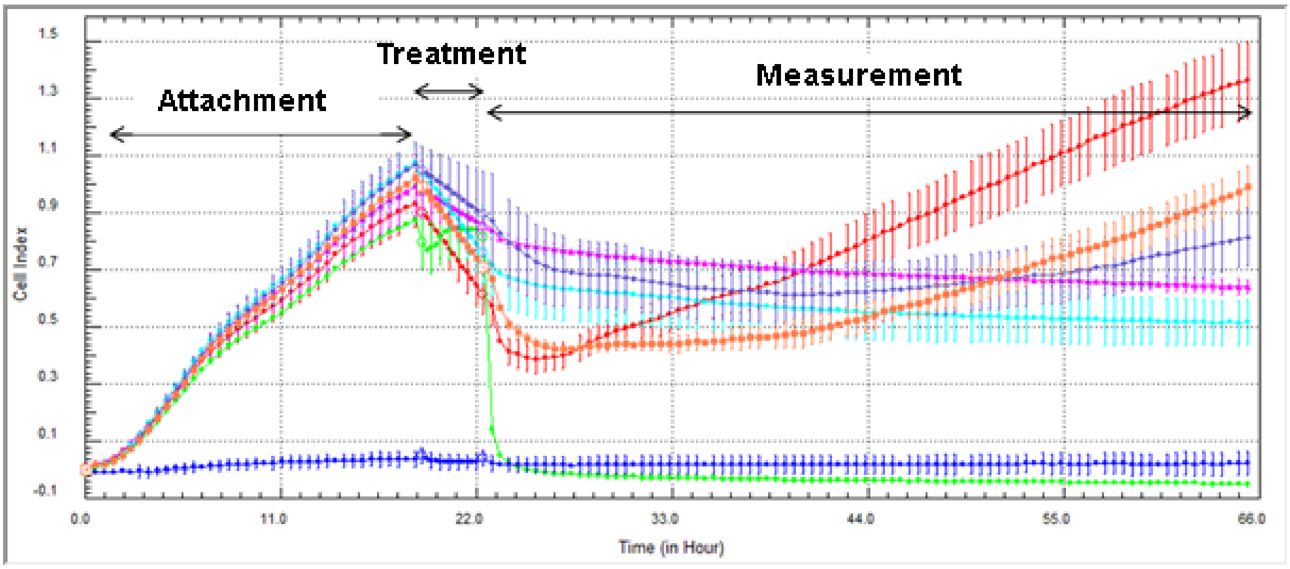

B

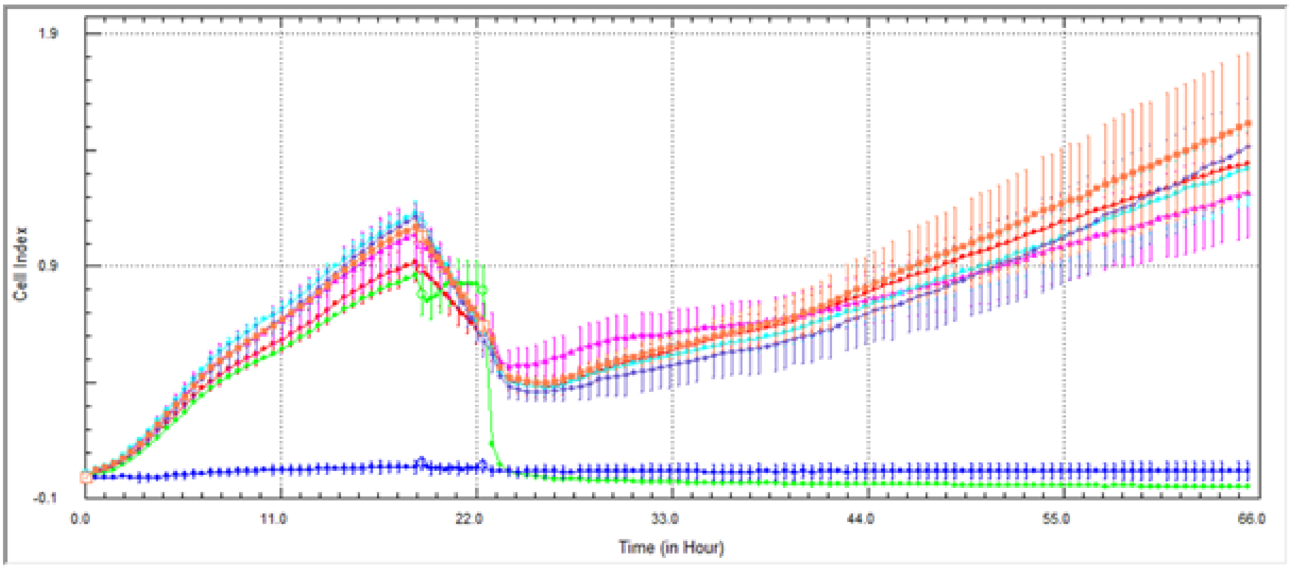

Supplementary Figure 1

Representative output of an RTCA experiment. Epithelial cells were allowed to attach to the bottom of the wells for 20 hours (**Attachment**). Medium was then aspirated and the cells were treated with 100 µl of the peptides dissolved in PBgluc (**Treatment**). After 3 hours of incubation at 37 °C, the peptide solution was removed and 200 µl of CKM was added to the cells. Subsequent real-time measurements of impedance were done with the xCELLigence System RTCA HT Instrument (ACEA Biosciences) for 72 hours (**Measurement**). The cell index at the beginning of the measurement after treatment was considered as 100% and values measured at 40 hours are expressed in Figures 3 and 4. A: treatment with a cationic NCR. B: treatment with an anionic NCR. Medium without cells (dark blue, baseline); Cells treated with primycin (green); Cells treated with NCRs at 0 (red), 12.5 (orange), 25 (medium blue), 50 (light blue) and 100 (pink) µg/ml. Notice that after primycin treatment cells cannot attah to the bottom of the cells because they lost their viability.
